# Clinical Lecture on Cerebral Symptoms Independent of Cerebral Disease

**Published:** 1861

**Authors:** Chas. West

**Affiliations:** Physician to the Hospital


### CLINICAL LECTURE ON CEREBRAL SYMPTOMS INDEPENDENT OF CEREBRAL DISEASE.

DELIVERED AT THE HOSPITAL FOR SICK CHILDREN, JAN. 26, 1861.

By CHAS. WEST, M. D., Physician to the Hospital.

The first set of conditions in which I have observed anxiety excited among non-professional persons, and occasionally among medical practitioners also, is when marked impairment of the intellectual powers, or marked alteration in the disposition, follows on convalescence from some serious illness, such illness having usually been of a febrile character, and probably having been typhoid fever more frequently than any other disorder.

Now, with reference to all these cases, it must be borne in mind that the younger the child, and the humbler its acquirements before the attack, the more far reaching will be the apparent results of any such illness. The disorder that befell the child who had not yet learned to speak, will postpone the attempt for months. It will reduce the little prattler, who had used its newly-acquired powers for a few months, to silence for several weeks, while it will manifest its effects in the older child by taciturnity, apathy, and indifference to its ordinary pursuits.

These effects are far more striking in the child than in the adult, not only because a similar impression produces a far deeper influence on the more fragile than on the stronger being, but also because the processes of growth and development, complete in the adult, are still in progress in the child, and after their interruption by disease, provident Nature cares first for the essential—the repair of the body—and does not till afterwards expend her energies in restoring the vigour of the mind.

Moreover, I remember no instance in which typhoid fever, or any other disease of which cerebral disturbance has not constituted a main feature, has been followed by permanent enfeebling of the mind, or by the development of disease of the brain, save indirectly by the production of tuberculosis and the subsequent supervention of tubercular hydrocephalus. It is, then, towards this point that attention should be mainly directed, and if the general nutrition is carried on well, no temporary dulling of the mind, no waywardness of the temper need make you falter in your assurance that time will set all right again.

At various epochs of development, symtoms not unfrequently appear which excite apprehension of cerebral disease, such symptoms consisting chiefly in altered disposition, in intellectual dullness, in irritable temper; but we shall, I think, usually interpret their meaning rightly by observing two things. They are moral and intellectual, much more than physical, in their nature, and they either coincide with, or follow, some definite stage of bodily development, such as the second dentition, or, in after age, the first efforts at pubescence, while, moreover, they are too variable, both in kind and degree, to tally with the indications of any definite organic disease of the brain itself.

Similar symptoms occur sometimes as the result of overtasking of the mental powers; and this not always by work excessive in itself, but excessive with reference to the then condition of the child, who may at one time be unequal to the amount of work which, a few months before, it was able easily to accomplish. The nature of the child's studies, too, sometimes overtasks its powers even more than the number of hours devoted to them, and, in such circumstances, the mere altering their nature, even though unaccompanied with any diminution in the time occupied by them, will often suffice to set matters completely right.

It is usually in connection with the development of the system which accompanies the second dentition that we meet with the severe neuralgic headaches of dyspeptic children. The history of such cases is usually something of the following kind: A child, whose general health has never been robust, whose nutrition is but imperfect, whose bowels are sluggish, and liver inactive, has occasional attacks of pain in the head; the pain is, for the most part, referred to the forehead, is attended with some intolerance of light, with sense of sickness, and, in some instances, with vomiting. It is usually very severe, so that the child seeks for perfect quiet, for a darkened room, and often asks to go to bed, where the attacks often subside after a short sleep, in the course of a few hours, though sometimesit it is not until after a night's rest that they pass away completely. On the day after an attack, however, the child seems in his usual health, though slight, over-excitement, over-fatigue, or mental effort will reproduce the pain, as will any error in diet, or want of attention to the state of the bowels. Independently, too, of any exciting cause, the attacks are apt to come on spontaneously every fortnight or three weeks, and their frequent recurrence at length excites the apprehension of the relatives of the patient, who cannot believe but that such frequent returns of severe suffering must needs imply the existence of formidable disease.

It is not easy to say how long, in any instance, these symptoms may continue. They are obviously dependent on the state of the general health, with the improvement of which they diminish or altogether disappear. The period of their most common occurrence seems to point to some connection between them, and the general state of the system which accompanies the evolution of the teeth; but they do not seem dependent, as some convulsive affections of infancy and childhood appear to be on the local irritation of one or two teeth in particular, ceasing as they pierce the gum. If I were to seek for an analogy, I should find it rather in that condition of the general health which in the girl at puberty accompanies the difficult and tardy development of the menstrual function, and which improves when that process is complete, and the function fully and regularly established. In confirmation of this view, I may add, that while in the boy it is very unusnal to observe this headache occur at the period of puberty, in the female sex the same kind of suffering not seldom recurs at that epoch, and sometimes, as we all know, continues in the woman even during the whole time of sexual activity, each recurring menstrual period being attended by intense headache, with much gastric disorder, though perhaps with little uterine pain, sometimes, indeed, with no pain at all referred to the sexual organs.

Pain in the head, accompanied with more or less intolerance of light, sickness, and constipation, are, as I scarcely need remind you, the symptoms that ordinarily usher in tubercular hydrocephalus; and though the knowledge of the public is inaccurate, they yet have a vague notion that such as these are the ordinary accompaniments of water on the brain, and they will come to you for a solution of their doubts, and a relief of their apprehensions. Now, I confess that, in many instances, the off-hand answer which is most desired is impossible; while, unluckily, there is no infallible criterion by which the caution suggested by wise observation can be distinguished from the hesitation of untaught inexperience.

Anyhow, it is worth while to have impressed on your mind the fact that the symptoms I have just enumerated do not *always* have so grave an import as commonly belongs to them, and that it is worth while to pause in every case before assigning to them their most untoward meaning.

These symptoms are for the most part observed a little later in life than the age at which tubercular hydrocephalus is most frequent, and they usually coincide with the progress of the second dentition. Further, although they are generally observed in children whose digestion has been liable to frequent disorder, and who are comparatively ill-nourished, the headaches are not preceded by that progressive loss of flesh or deterioration of the general health, which, in many cases, though confessedly by no means in all, precedes the development of acute hydrocephalus. Moreover, it will often be found that the headaches have continued to recur at intervals for weeks or months before the patient came under your notice, that yet no progressive deterioration has been taking place in the child's condition, that the headaches are not more intense, that no fresh symptom of cerebral disorder has supervened; but that such as they were months before, such they continue still. The attacks of headache themselves present some characters by which they may be distinguished from those whose import is more formidable. They are not attended with any remarkable increase of heat of head, nor with any marked pulsation of the carotids. The pulse, though quickened, is rarely unequal or irregular; the bowels, though constipated, answer comparatively readily to aperients; actual vomiting is unusual, and the nausea even rarely outlasts the seizure. Stimulants, too, not unfrequently relieve the pain, instead of aggravating it, and though excitement or over fatigue may have brought on the attack, yet a tale that interests or arouses the child, or any occurrence which brings to it much pleasure, often dissipates the pain completely; while, moreover, the spirits in the intervals become as buoyant as ever, and continue so until the next attack, a marked contrast to the anxiety and depression which often precede for weeks or months the onset of the acute stage of tubercular hydrocephalus.

Pure neuralgia, like the neuralgic headache of the adult, though less common than this sick headache, if so I may term it, is yet sufficiently frequent to need being borne in mind, when the question arises as to the import of symptoms supposed to indicate cerebral disease. The headache in these cases is usually frontal, and care is therefore the more necessary, since there is no doubt but that continued frontal headache is a very frequent accompaniment of organic disease of the brain, and especially of tubercle of that organ. The severity of the pain, the suddenness of its onset, the completeness of its cessation, the fact that its recurrence for days is attended by no progressive deterioration in the patient's condition, all furnish a clue to its real nature. Moreover its supervention after exposure to malaria, and a certain ill-marked periodicity in its return, are circumstances which will serve in many instances, no less than the absence of other signs of disease of the brain to guard against, too hasty, or too positive an expression of an unfavorable prognosis.

There is besides another large class of cases, in which the occurrence, not merely of vague indications of cerebral disorder, but of violent convulsive attacks, excites alarm, proportionate rather to the formidable nature of the symptoms, than to the importance of the cause on which they really depend.

One of the most remarkable instances of this class which I have ever met with, was furnished by a little girl, 6½ years old, who suffered much from ascarides, on account of which she came under my care on September 16, some years ago. She had benefited somewhat by aperient and other treatment, when on the evening of October 7 she complained of pain in the chest; and at eleven in the evening, having then been some hours in bed, she was seized with a fit, which did not entirely cease till two o'clock the next morning, when she fell asleep, and awoke apparently well about seven o'clock. At nine a second fit occurred, which lasted till eleven; and from that time till the afternoon of the eleventh, fits continued to recur, having at no time a longer interval between them than six hours.

The fits were not quite like ordinary epilepsy; they began with slight rolling of the eyes, and twitching of the mouth, to which succeeded very rapid movements of the throat as if in efforts at deglutition, and these movements became soon accompanied by a sound somewhat between that of a sob and a hiccough, and not unlike the sob of a hysterical patient. During this time the pupils became dilated, the pulse feeble, and the child partially insensible, but she never became absolutely so, and complete consciousness returned long before spasmodic movements ceased, or the convulsive sound in the throat subsided; the child, when asked if she was in pain, pointing to her throat. Very active purgation, during which a large quantity of ascarides was expelled, was followed by the cessation of the convulsive attacks; but for nearly thirty-six hours afterwards, the child complained of pain in the throat, and swallowed difficultly and with a sort of convulsive effort.

Now, how formidable soever the symptoms of disorders of the nervous system may be, it is a rule, subject, I think, to a very few exceptions, that the more anomalous such symptoms are, the greater is the probability of their depending not on grave organic disease, but on some excentric and generally removable source of irritation. In infancy we are so aware of this fact, that when convulsions occur, our first impulse always is to seek in some excentric disturbance for their occasion. I think, however, that we are too apt to forget that this peculiar susceptibility continues, to a great degree, through all the years of childhood.

In the case just related, the presence of a large quantity of ascarides produced a succession of violent convulsions, which ceased permanently on their complete expulsion. Sometimes, however, the irritation occasioned by the existence of worms in the intestines has more of an intermittent character coinciding with the movements of the parasite, and subsiding for a time on the expulsion of a lumbricus, or the returning quiescence of the tape-worm.

A little boy, 7½ years old, nervous, excitable, ill-managed, and liable to night-terrors, woke at four one morning, crying; the cries were soon succeeded by an attack of convulsions, which differed from an ordinary epileptic seizure, it being attended by a less profound degree of insensibility than ordinarily accompanies it. For a few weeks the child continued peculiarly excitable, wayward, and fretful, two more convulsions then occurred, soon after the last of which he voided a large lumbricus. This occurrence was followed by a marked improvement, which lasted for a few weeks. The old symptoms then returned, followed by another convulsion, and then another lumbricus was expelled, once more with an amelioration of the symptoms, and a second cessation of the fits. Whether the improvement was permanent, or whether the former symptoms returned, I cannot say, as I lost sight of the patient, but enough has been told of the child's history, to show the connection between the intestinal irritation produced by the worms, and the violent disturbance of the nervous system.

Though I have given these illustrations of nervous symptoms dependent on the presence of worms, and it would not be difficult to add to their number, it is yet not out of place to caution you against assuming the presence of worms merely because such an assumption furnishes you with the readiest clue to the meaning of certain symptoms of cerebral disorder which you do not find it easy at once to account for. I have seen the early stages of acute hydrocephalus referred to worms, just as I have seen the indisposition which ushered in typhus fever referred to them, without the slightest evidence of their existence, but of mere mental indolence on the part of the practitioner which indisposed him from careful observation or thoughtful consideration, and led him to take up with the hypothesis which served most readily to relieve him from the task of watching and reflection.

When symptoms of cerebral disorder depend on the presence of worms, there has almost always been ample evidence of their existence before the nervous system became gravely disturbed by them. In the few exceptions to this rule it may be asserted almost positively that their appearance will follow nearly at once on the cerebral disorder which awakens our anxiety and the alarm of the patient's friends. Moreover, the observation I have just made as to the influence which the anomalous character of symptoms fairly suggests, may be borne in mind in all doubtful cases with much advantage; and the question deliberately propounded to yourselves, "Of what definite disorder of the brain or nervous system are these symptoms; or in what respects are they anomalous and discrepant?" will often save you from many grave errors and many useless regrets.

We are all so familiar with the occurrence of convulsions during the first dentition, that in infancy the risk is rather of the grave disease to which they are possibly due being overlooked, than of the influence of teething in their production being underrated. On the other hand, the occasional share of the second dentition in exciting epileptic or other convulsive seizures is too little borne in mind, and a graver prognosis than the event justifies is sometimes expressed in consequence.

In a little work on Dentition,* published some years ago by my friend and former colleague, Dr. Ashburner, there are many cases related illustrative of this fact. Of these, I will select the following:

* On Dentition, etc. 12mo. London, 1834, p. 97.

"A boy, twelve years of age, was cutting the second or posterior permanent molars of the upper jaw before those of the lower, and the process was accompanied by twitchings of various parts of the body. At last he became affected with chorea. Being a very nervous lad, if any notice were taken of him, he would quite involuntarily make the most extraordinary faces, and contort his body into various attitudes that appeared to be most difficult and painful. This chorea continued for three months, during which time a variety of medicines were swallowed. At last he fell into an epileptic fit, struggling much, foaming at the mouth, and grinding the teeth. I thrust my fore-finger along the inside of his cheek, and found a hard, cartilaginous space on each side, behind his first molar tooth. I succeeded in gashing these parts. He uttered a scream and fell out of his fit, becoming quite sensible, nor had he a recurrence of his chorea."

So sudden and complete a cessation of symptoms on the removal of the mechanical irritation produced by the pressure of a tooth is decidedly an unusual occurrence. In by far the greater number of instances, the symptoms of disorder in the nervous system do not admit of being cut short thus suddenly and decisively; they depend not simply on the local irritation produced by one particular tooth, but, like the headachs which I spoke of at the commencement of this lecture, they are the result of the disturbance of the nervous system, to which the whole process of development has given rise, just as, in later life, the hysteria of the young woman is connected with the imperfect accomplishment of sexual function, and is not removed by a single occurrence of menstruation.

If this class of cases may be referred the history of a boy between eleven and twelve years old, whom I saw some years ago. While, apparently, in good health he was atacked by a fit one morning, in which his head was drawn to the right side. His face was distorted, especially his right side, and his right arm was in much more violent movement than his left. This fit was not preceded nor followed by sleepiness or headache, nor was it attended by any loss of consciousness, and the same characters were observed in all his subsequent seizures except one, which—the third in order of occurrence—was followed by temporary delirium for five or ten minutes. With the exception of the first and third of these attacks, all the others ceased in a minute, and the instant the spasmodic movements subsided, the boy resumed his former occupation, as if nothing had happened. Only once did more than one seizure take place in the course of twenty-four hours; but the intervals between two attacks were very irregular; sometimes eight days had passed without an attack, while, at other times, they recurred daily for three days; and, altogether, between the beginning of July and the end of December, more than fifty occurred, with no alteration of their character or increase in their severity, while neither the boys' health nor his intellect appeared to have suffered in any respect from the affection. One point observed with reference to them—which, I may add, is often noticed in cases of epilepsy—was, that they almost invariably happened soon after rising in the morning, generally between the hours of seven and nine o'clock.

At first, he was actively purged, but with no effect; he then took the valerianate of zinc likewise fruitlessly; and then, as he was cutting the second molar tooth in the right side of the upper jaw, and as there was much tenderness of the gum over it, the gum was freely lanced, and all treatment was suspended. No amendment followed this measure, and the boy was, therefore, put for a time on the nitrate of silver, but with no results.

The idea which had been entertained of the possible connection of the attacks with the process of dentition appeared to me still to be the most probable, and for the following reasons: That, in spite of the continuance of the attacks, there had been no such deterioration in the boy's condition as might have been expected if they had been dependent on cerebral disease; that no impairment of powers over the affected side had followed the attacks, while such an occurrence would have inevitably occurred if the attacks had been dependent on tumour or tubercle in the brain; that headache was completely absent, and all the general functions were perfectly well performed; while, further, the anomalous character of the attacks themselves seemed still further to point to some excentric source of irritation.

On looking into the boy's mouth, the teeth in the upper jaw were observed to be very crowded and overlapping each other; the first molar on the left side was decayed, the second molar was still beneath the gum. I advised the extraction of the decayed tooth, and lancing the gum over the second molar, while all medical treatment besides should be discontinued. Before these measures were adopted, the boy had six consecutive attacks in the course of one day; two of them being very severe, and attended like ordinary epilepsy, with loss of consciousness. The extraction of the decayed tooth was followed by immediate diminution in the convulsive attacks, both in point of frequency and severity, and the improvement lasted for nearly two months. The second molar tooth was then partly through the gum, when a succession of seizures, some of them accompanied by loss of consciousness, once more took place. Free lancing of the gum was succeeded, as before, by immediate improvement, and not only so, but the tooth being now quite through the gum, all spasmodic movements of the arm ceased, and occasional slight twitchings of the face were the only relics of symptoms which had seemed so formidable.

One caution, which this case suggests, I cannot forbear to offer, although it is not immediately connected with the subject of this lecture. It relates to the possibility of attacks of this nature, and still more of those of a more decidedly epileptic character, becoming permanent as the mere result of frequent recurrence, and this quite independently of the existence of any organic mischief in the brain. Thus, I have seen instances in which the convulsions that originated in whooping cough, have outlasted the disease that occasioned them, and the fits which at first were due to some error in diet, reproduced again and again by similar though perhaps smaller indiscretions in the same respects, until they became at length habitual. Each fit, too, leaves the child more predisposed to its occurrence, and from slighter and still slighter causes; so that never is the old Latin adage, *Obsta principiis*, more important than in the case of the convulsive diseases of early life.

In the majorisy of cases of chorea no diagnostic difficulty arises; the irregular movements of the limbs, the strange grimaces and gesticulations, sufficiently characterize the disease. Now and then, however, though the movements of the limbs are very slight, or partial, the speech is much affected, and the child, partly, perhaps, in consequence of the difficulty in communicating its ideas, becomes dull, low-spirited, and apparently sullen, and apprehensions are thus excited lest these unusual symptoms should betoken disease of the brain.

A boy, aged 8½ years, whose health had always been delicate, though he had suffered from no special illness, seemed feebler than usual when he returned from school for his Christmas holidays, and his mother's anxiety was excited all the more with reference to his indisposition from the circumstance that his father had died of consumption. In the course of a fortnight from his return home, it was noticed that power over his right hand was much impaired, and three days after, power over his right leg failed also, and his speech became at the same time much affected. The speech grew rapidly more and more unintelligible, the boy stumbled when walking, and occasionally fell, and there were slight twitchings of the muscles of the right arm and leg, though these were at no time remarkable, and the face was never distorted. With the progress of these symptoms, too, the boy, never very quick, had become peculiarly dull and apathetic, so that it was not to be wondered at if the attention of his friends became fixed on those symptoms which were of the gravest import, and were apprehensive lest they should betoken disease of the brain.

Close inquiry, however, elicited facts that seemed to warrant the more hopeful view which the event of the case fully justified. There was no complaint of headache, there had been no sickness; the pulse, though feeble, was not all irregular; the tongue was protruded straight; the pupils were equally dilated, and equally susceptible to the influence of light; the movements, which ceased completely during sleep, were unaccompanied by any rigidity or contraction of the muscles of the affected limbs, and close observation discovered that though extremely slight on the left side, the irregular movements did affect both sides of the body. In a week or two more the affection of the left side became quite as marked as that of the right, deglutition, too, as well as speech, became affected, and the anomalous symptoms merged into those characteristic of an ordinary attak of somewhat severe chorea, from which under general tonic treatment the boy recovered, the brightening of his mind preceding, as I have seen it do in other instances, the amendment of the purely choreic symptoms.

I have seen, two or three times, a peculiar spasmodic affection in children which, though I believe it to be of no serious moment, yet has attracted attention, and excited anxiety by its singularity. It consists in the extremely frequent occurrence of involuntary sighing respiration: the child, who seems otherwise in tolerable health, and who follows his usual occupations and amusements, though listlessly and languidly, fetching a deep sigh almost every second or third inspiration, with a most painful and depressing monotony. All day long the sighing continues, ceasing, indeed, at night when sleep is undisturbed, but beginning again in the morning. No external condition appears to check, or indeed much to modify it; it is usually worse when the child has been long without food, but eating does not put a stop to it, and the child sighs, as if most woe-begone, between each few mouthfuls that he takes. Once I saw the sighing followed by a causeless attack of difficult breathing similar to the nervous dyspnœa of a hysterical patient, but usually from beginning to end the sighing continues the only symptom of illness. The attack comes on gradually, and subsides in the same manner. In the instances which I have seen, however, it did not continue for much above ten days with such distinctness as to force itself upon observation, but abated by degrees under the employment of mild tonics, and of attention to the digestion, which usually is more or less disordered.

I have never seen it associated with other symptoms pointing to disorder of the brain, nor have I known it issue in any graver ailment, but its singularity has excited fears which perhaps this mention of it may serve to allay.

I must not close this lecture without some reference to a very important class of cases, to which, indeed, I hope on a future occasion to call your special attention, where the loss of power over some of the muscles raises the suspicion that the brain is the seat of serious disease. In the adult, we are familiar with the occasional occurrence of partial paralysis, especially of fascial palsy, independently of cerebral disease. We can therefore the more readily understand how a similar occurrence may take place in the more sensitive and delicate frame of the child without any abiding disease of the nervous centres. Those slow processes of disorganization which, affecting the brain, in the adult produce palsy, sometimes of one part, sometimes of another, so that we look on the impaired power of movement as the attendant commonly on some grave disease, and the harbinger of its fatal issue, are of extreme rarity in the child. Tumors, almost invariably tuberculous, within the brain, or disease of the spinal column extending from without inwards, until by its advance the cord itself becomes implicated, are almost the only conditions in which paralysis in childhood depends upon mischief seated in the nervous centres. In the young, the loss of power is indeed uncertain in its issue, as far as the complete recovery of the patient is concerned; it is grievous in its results, for it often leads to permanent deformity; but it is very rarely that it need excite apprehension with reference to life, or health, or intellectual power. In the grown person, the import of paralysis is commonly of the gravest kind, subject, indeed, to exceptions, of which fascial paralysis furnishes the most frequent illustration. We are bound, indeed, to scrutinize each case most carefully; but in the grown person, our inquiry commonly resembles that which the Judge puts to the condemned criminal when he asks him whether he has anything to say why sentence of death should not be passed upon him. In the child, care, too, is needed not to overlook some peculiarity in the case which may attack to the loss of power a worse than is wonted to significance; but yet you will do well to remember that life is very rarely threatened by paralysis in early life; that organic disease is the rare exception, not the ordinary occurrence. It will save you much useless anxiety, and your patient's friends much needless sorrow.

*Medical News.*

